# Have patient pathways for penetrating chest injuries improved since designation as a London major trauma centre?

**DOI:** 10.1186/1757-7241-21-S1-S21

**Published:** 2013-05-28

**Authors:** JR Pallett, J Olding, T Hurst, M Tunnicliff, JW Keep

**Affiliations:** 1 Kings College Hospital, London, UK

## Background

King’s College Hospital NHS Foundation Trust was designated a Major Trauma Centre for the South East London Trauma Network in April 2010. Penetrating trauma accounts for a high proportion of the Trauma Team caseload at this centre and various initiatives have been made to improve the quality of the major penetrating trauma pathways.

## Methods

A retrospective cohort study of penetrating chest trauma cases seen by the Trauma Team from April 2010 and April 2011 was compared. Statistical analysis of unpaired means was calculated with the *z*-test and two-tailed p-values.

## Results

25 cases from April, May and June 2010 (100% male, mean age 28.6 years) and 28 cases from April, May and June 2011 (100% male, mean age 24.1 years) were indentified. In 2010, mean time to CXR was 18.86 minutes, 95% CI (12.97, 24.75) and mean time to CT was 93.10 minutes (61.27, 124.93). In 40% of cases use of *eFAST* was documented. In total, 91.3% underwent CT scanning of the thorax. In February 2011, a Standard Operating Procedure (SOP) was introduced along with pre-registration of patients prior to arrival, use of a designated trauma CT scanner and increased training of *eFAST*. In 2011, mean time to CXR was 10.45 minutes, 95% CI (7.55, 13.35) and mean time to CT was 46.94 minutes, 95% CI (26.36, 67.52). In 54% of cases use of *eFAST* was documented. In total, 60.7% of cases underwent CT scanning of the thorax. Comparing this data over time, both time to CXR (p=0.012) and time to CT (p= 0.000024) have significantly decreased along with increased utilisation of *eFAST* and reduced use of CT scanning in accordance with the SOP.

## Conclusion

In the intervening year as a Major Trauma Centre, there is quite strong evidence (p<0.05) for CXR and very strong evidence (p<0.0001) for CT, that time to diagnostic imaging has significantly improved. The introduction of an SOP incorporating physiological parameters and zone of chest injury along with increased use of *eFAST* has contributed to a reduction in the use of inappropriate CT scanning in accordance with recent evidence-based guidance.[1]
